# Study of Cinobufagin as a Promising Anticancer Agent in Uveal Melanoma Through Intrinsic Apoptosis Pathway

**DOI:** 10.3389/fonc.2020.00325

**Published:** 2020-04-02

**Authors:** Leilei Zhang, Xiaolin Huang, Tao Guo, Huixue Wang, Haiyan Fan, Li Fang

**Affiliations:** ^1^Department of Ophthalmology, Ninth People's Hospital, Shanghai Jiaotong University School of Medicine, Shanghai, China; ^2^Shanghai Key Laboratory of Orbital Diseases and Ocular Oncology, Shanghai, China

**Keywords:** cinobufagin, anticancer agent, uveal melanoma, apoptosis, MMP

## Abstract

Uveal melanoma (UM) is the most common primary intraocular carcinoma in adults. Cinobufagin, secreted by the Asiatic toad *Bufo gargarizans*, is a traditional Chinese medicine, widely used in tumor treatment. Here, we explored the potential antitumor function of cinobufagin and investigated its biochemical mechanisms in UM cells. The antitumor potential of cinobufagin was determined via cell viability, cell cycle, and apoptosis assays. Colony formation assays confirmed that cinobufagin exerted potent antitumor activity in a dose-dependent manner. We found that cinobufagin could induce cell apoptosis and upregulate the expression of cleaved caspase-3, cleaved poly(ADP-ribose) polymerase (PARP), and cleaved caspase-9 *in vivo* and *in vitro*. In addition, after treatment with increased concentrations of cinobufagin, the intrinsic mitochondrial apoptosis pathway was also activated, which was demonstrated by increased cell apoptosis with increased expression of Bad and Bax, decreased expression of Bcl-2 and Bcl-xl, and reduced mitochondrial membrane potential (MMP) in OCM1 cells. Taken together, the results of this preclinical study suggest that cinobufagin can both inhibit cell survival and induce cell apoptosis in a dose-dependent manner in UM cells, which provides new insights into the biochemical mechanism of cinobufagin and its potential as a future chemotherapeutic agent for UM.

## Introduction

Uveal melanoma (UM) represents the most frequently occurring primary intraocular malignant carcinoma in adults, with an annual incidence rate of 5.1 cases per million. Lesions are situated either in the choroid (90%), ciliary body (6%), or iris (4%) (1). Currently, the main treatment of UM is surgery, radiotherapy, and even enucleation. However, the long-term survival rate of uveal melanoma is relatively low due to the risk for liver metastasis. Thus, early detection and treatment of small lesions play a crucial role in achieving not only local disease control and vision preservation but also the possibility of preventing metastasis and improving overall patient survival (1, 2).

Traditional Chinese medicine (TCM) provides valuable resources for the treatment of cancers (3). With higher efficiency, weaker side effects, easier access, and better quality of life outcomes, TCMs are widely used in clinics (4). Recently, in the United States and Europe, TCM has been widely used as a complementary and alternative treatment (5).

Chan-su is a universally known TCM among some Asian countries, which is derived from the dried secretion and parotid glands from the skin of the Asiatic toad *Bufo gargarizans* (6). Cinobufagin (CBG) is a primary and active component of Chan-su, which has potential anticancer properties (7). Researches have demonstrated that cinobufagin showed antitumor effect in gastric cancer (8), lung cancer cachexia (9), breast cancer (10), osteosarcoma (11), and pancreatic cancer.

Preliminary studies have also demonstrated that CBG could suppress cell growth and induce apoptosis via the Notch pathway in osteosarcoma cells. The expression of Notch-1, Hey-1, Hes-1, and Hes-5 was significantly downregulated after treated with cinobufagin in osteosarcoma (OS) cell lines. In addition, the activation of Notch pathway could attenuate CBG-induced apoptosis (11). Another report showed that cinobufagin could induce cell apoptosis through the intrinsic, mitochondrion-dependent apoptosis pathway via the aggregation of reactive oxygen species (ROS) and the loss of mitochondrial membrane potential (ΔΨm) in FOB1.19 and U2OS osteosarcoma cells (12). In U266 human multiple myeloma cells, cinobufagin possibly exerted its antitumor effects via the activation of c-JUN N-terminal kinase (JNK), extracellular signal-regulated kinase (ERK), p38, mitogen-activated protein kinase (MAPK), and caspase-3 mediated through ROS (13). Cinobufagin inhibited the cell growth and triggered apoptosis via the alternation of the expression of Bax and Bcl-2 in human breast cancer MCF-7 cells (10). However, the antitumor activities and biochemical mechanism of cinobufagin in uveal melanoma are still elusive and need to be further elucidated.

## Materials and Methods

### Chemicals and Reagents

Cinobufagin was purchased from Anhui Jinchan Biochemical Co., Ltd. (Anhui, China). Cell counting kit-8 (CCK-8) was purchased from Dojindo (Kumamoto, Japan). The primary antibody against β-actin was purchased from Sigma (St. Louis, MO, United States). Primary antibodies against caspase-3, caspase-9, Bcl-2, and PARP were purchased from Abcam (Cambridge, United Kingdom).

### Cell Culture

The human UM cell line OCM1 was cultivated in Dulbecco's modified Eagle's medium (DMEM) containing 10% fetal bovine serum (FBS) and 1% antibiotics (penicillin, 100 U/ml; streptomycin, 100 μg/ml). These cells were placed in a humidified environment at 37°C with 5% CO_2_ and 100% humidity. The culture medium was replaced every 3 days.

### Cell Viability Assay

OCM1 cells were suspended at a final concentration of 5 × 10^3^ cells/well and cultivated in triplicate in a 96-well plate. The viability of cells was measured via the CCK-8 assay at the indicated time points. Then, CCK-8 (10 μl) was added to each well-containing a 100-μl mixture of culture medium. The plate was incubated for 1.5 h at 37°C. Viable cells were counted by absorbance measurements at 450 nm using an automated microplate reader (Tecan Sunrise, Austria). All experiments were performed in triplicate.

### Flow Cytometry

Cells were collected and fixed in 70% ethanol, treated with 300 μg/ml RNase A (Sigma-Aldrich, St. Louis, MO, United States), and their nuclei were stained with 10 μg/ml propidium iodide (Sigma-Aldrich, St. Louis, MO, United States). The stained nuclei were detected by a FACSCalibur cytometer. The data were analyzed by ModFit software. The apoptosis assay was performed using an Annexin V-FITC apoptosis detection kit (Sigma-Aldrich, St. Louis, MO, United States). Cells were washed twice with phosphate-buffered saline (PBS) buffer and then resuspended in 1 × binding buffer at a concentration of 1 × 10^6^ cells/ml, and 5 μl of Annexin V-FITC conjugate and 10 μl of propidium iodide (PI) solution were added to each 500-μl cell suspension. Cells were then stained with Annexin V-FITC/PI for 10 min at room temperature. Stained samples were analyzed via FACS Calibur, and the concrete apoptosis rate was determined using FlowJo software (11).

### Western Blotting

Proteins were extracted via protein lysis buffer. Lysates were centrifuged at 12,000 × g at 4°C for 5 min, and the supernatants were collected. The protein concentration was assessed via the bicinchoninic acid protein assay kit. Cell lysates containing 40 μg protein were separated on a 10% sodium dodecyl sulfate–polyacrylamide gel electrophoresis (SDS-PAGE) gel and then transferred onto polyvinylidene difluoride (PVDF) membranes (Millipore, United States) using a Trans Blot Turbo. Membranes were then blocked in a solution of Tris-buffered saline containing 0.05% Tween-20 and 5% skimmed milk for 60 min at room temperature. Primary antibodies were incubated overnight at 4°C. The primary antibodies used were anticleaved caspase-9 (1:1,000), anticleaved caspase-3 (1:1,000), anti-PARP (1:1,000), anti-Bcl-2 (1:1,000), anti-Bcl-xl (1:1,000), anti-Bad (1:1,000), anti-Bax (1:1,000), and anti-β-actin (1:5,000). Horseradish peroxidase-conjugated secondary antibodies were incubated with the membranes for 2 h at room temperature. Membranes were finally developed via an enhanced chemiluminescence substrate (11).

### Mitochondrial Membrane Potential Measurement

The ΔΨm was evaluated via a commercial mitochondrial membrane potential assay kit with JC-1 (Invitrogen, Carlsbad, CA, United States). Cells were treated with the suggested concentrations of cinobufagin (0, 0.1, 1, and 3 μM) in six-well-plates for 24 h and stained with JC-1 for 15 min at 37°C in the dark. Then, the cells were then harvested, washed twice with PBS, and resuspended in 1 ml PBS for subsequent flow cytometry analysis.

### Tumor Xenograft Model in Nude Mice

OCM1 cells (1 × 10^7^ in 200 μl per injection) were subcutaneously injected into the right flanks of 3-week-old thymic nude mice (*n* = 20). On the ninth day, when the tumor size was ~100 mm^3^, the nude mice were randomly divided into four groups (*n* = 5), and experimental group 1 began receiving drugs via intraperitoneal injection of CBG at 5 mg/kg once a day for 10 days. Control group 1 was intraperitoneally injected with 0.5 mg/kg saline for 10 days. On the fifth day, 10 mice had tumors up to 300 mm^3^ in size, and experimental group 2 was injected with cinobufagin at 5 mg/kg once a day for 7 days. Control 2 group was untreated. The mice were housed under a controlled environment in a sterile facility. The tumor volume was measured every 3 days with calipers. Tumor volume was calculated using the formula: 0.5 × length × width × width. After 30 days, the mice were killed; then, the tumors were removed and analyzed. The Animal Care and Use Committee at Shanghai Jiaotong Medical University approved the animal protocols.

### Total RNA Isolation, Reverse Transcription, and Quantitative Polymerase Chain Reaction

The tissues were immersed in TRIzol reagent (Invitrogen, Carlsbad, CA, United States) and ultrasonicated to extract the total RNA according to the manufacturer's protocol. Complementary DNA (cDNA) was synthesized using the PrimeScriptTM RT reagent Kit (TaKaRa, Tokyo, Japan). The concentration and purity of total RNA were spectrophotometrically determined by the optical density (OD) at 260 nm and the OD at 280 nm using a NanoDrop 2000 UV spectrophotometer (Thermo Scientific, United States) (14). The primers for caspase-3, PARP, Bcl-2, Bcl-xl, Bad, and Bax used for reverse transcription quantitative polymerase chain reaction (RT-qPCR) analyses are described in Supplemental Table 1. RT-qPCR was conducted using the Applied Biosystems 7500 Real-Time PCR System (Applied Biosystems, Irvine, CA, United States) and SYBR Premix Ex TaqTM (TaKaRa, Tokyo, Japan). The PCR for each gene was repeated at least three times for each sample, and data were analyzed by comparing the 2^−ΔCt^ values. Gene expression levels were normalized to *GAPDH* (14).

### Statistical Analysis

Statistical analysis was conducted via Prism 5 software (GraphPad Software, Inc., La Jolla, CA, United States). Data were shown as the mean ± standard error of mean (SEM). The significant difference between the treated and the control was tested by Student's *t*-test. The Kaplan–Meier method was used to estimate the survival curve and the log-rank test to generate statistical significance. *P* < 0.05 was considered as statistically significant. Each experiment in this article was repeated at least three times (15).

## Results

### Cinobufagin Exerted Potent Cytotoxic and Anticell Growth Activity Against Human UM Cells

**Figure 1:**
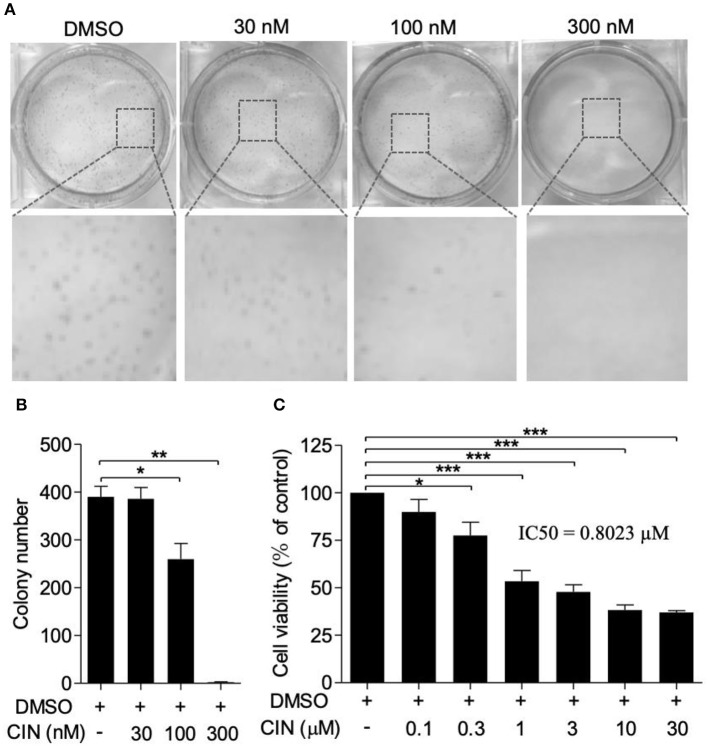
Cinobufagin inhibits OCM1 cell proliferation. **(A)** Colony formation assay was used to determine the inhibitory effect of cinobufagin (CBG) on OCM1 proliferation. Representative images of colonies from six-well plates by colony formation assay. OCM1 cells were treated with 0, 30, 100, and 300 nM CBG for 1 week. **(B)** Quantification of the colony number of OCM1 cells. **(C)** CCK-8 assay was used to detect the toxicity of CBG after treatment for 48 h on OCM1 cells. The IC50 was calculated to be 0.8023 μM. The results are expressed as the mean ± SEM of three independent experiments [*n* = 3, *P < 0.05, **P < 0.01, and ***P < 0.001 compared with the dimethyl sulfoxide (DMSO) control].

To explore the potential effect of cinobufagin on uveal melanoma cells, a colony formation assay was used to determine the inhibitory effect of cinobufagin. As shown in Figure 1A, cinobufagin had a significant inhibitory effect on OCM1 cells. After treatment with different concentrations (0, 0.03, 0.1, and 0.3 μM) of cinobufagin for 1 week, the number of colonies decreased in a dose-dependent manner (Figure 1B). In addition, the CCK-8 cell viability assay showed that the proportions of living cells were approximately 87, 77, 53, and 48% at concentrations of 0.1, 0.3, 1, and 3 μM cinobufagin, respectively. However, after treatment with 10 and 30 μM cinobufagin, the proportion of viable cells was only 37 and 35%, respectively (Figure 1C). These results demonstrated that cinobufagin effectively inhibited OCM1 cell survival. Here, the IC50 value of cinobufagin in OCM1 cells was 0.8023 μM after treatment for 48 h. In consideration of the toxicity of cinobufagin, we chose 0.3, 1, and 3 μM concentrations for the following experiments.

### Cinobufagin Arrested OCM1 Cell Cycle Progression

**Figure 2:**
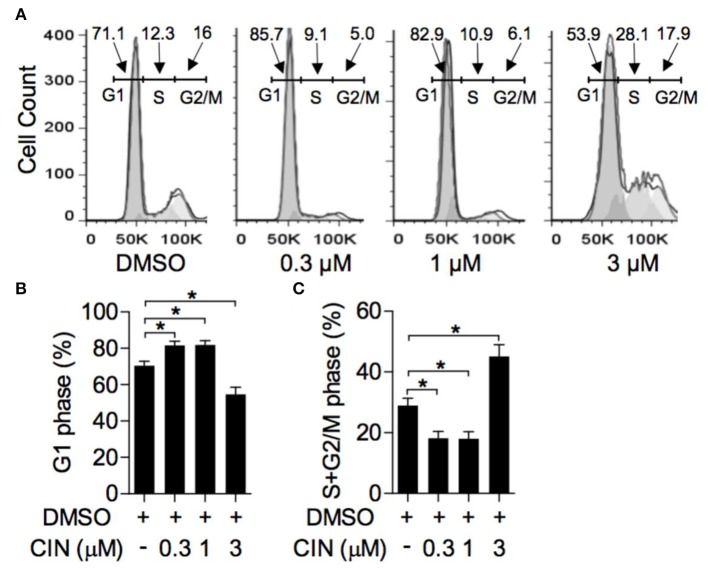
Cinobufagin induces cell cycle arrest of OCM1. **(A)** OCM1 cells were treated with 0, 0.3, 1, and 3 μM cinobufagin. After 24 h, OCM1 cells were collected and stained with propidium iodide (PI). Then, the cell cycle distribution was measured with flow cytometry. The percentages of cells in the **(B)** G1 phase and **(C)** S + G2/M phase were quantified. The results are displayed as the mean ± SEM of three independent experiments [*P < 0.05, compared to OCM1 cells incubated with DMSO control].

The impact of cinobufagin on OCM1 cell cycle progression was measured by flow cytometry. As shown in Figure 2A, after treatment with cinobufagin for 24 h in OCM1 cells, the percentage of cells in G1 phase significantly increased (Figure 2B). Correspondingly, the percentages of S and G2/M phase cells decreased (Figure 2C). In addition, cinobufagin arrested the cell cycle in the G1 phase in a concentration-dependent manner. It showed that cinobufagin induced cell cycle arrest in OCM1 cells.

### Cinobufagin Induced Cell Apoptosis and Alterations of Apoptosis-Related Proteins in OCM1 Cells

**Figure 3:**
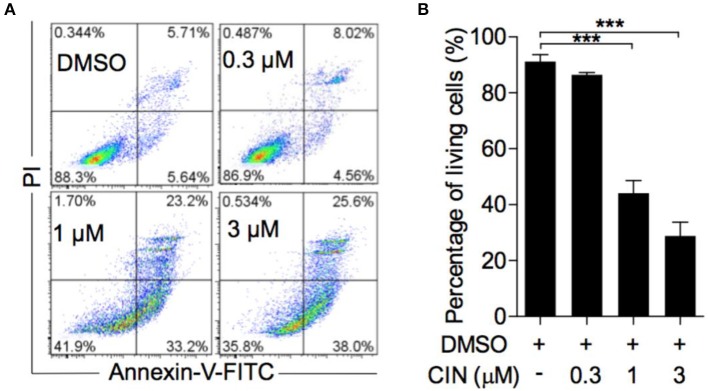
Cinobufagin induces OCM1 cell apoptosis. **(A)** OCM1 cells were treated with 0, 0.3, 1, and 3 μM cinobufagin (CBG) for 24 h. Then, the cells were stained with Annexin V-FITC/PI and measured by flow cytometry. The living cells were located in the lower left quadrant. **(B)** Quantification of living OCM1 cells. The results are expressed as the mean ± SEM of three independent experiments [***P < 0.001 compared with OCM1 cells treated with DMSO].

**Figure 4:**
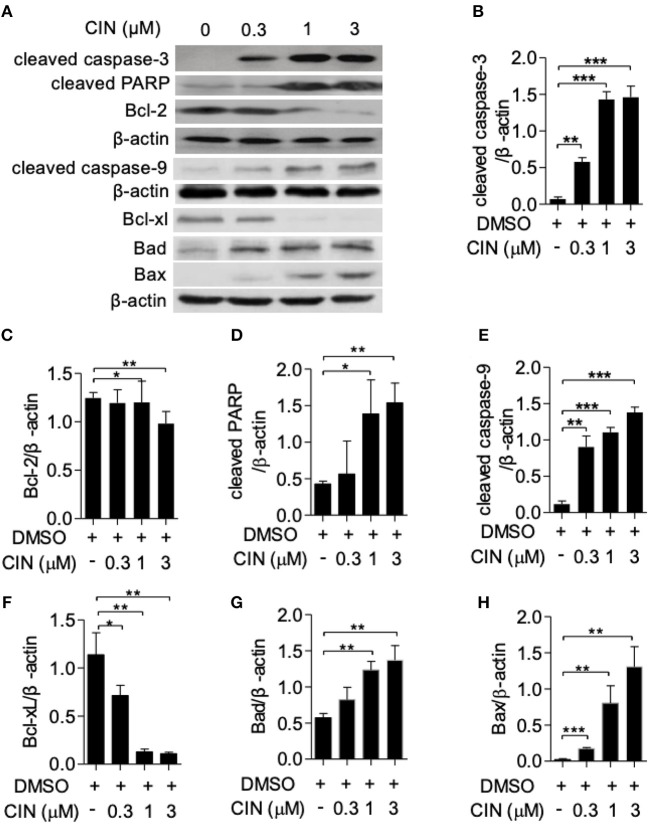
Cinobufagin triggers apoptotic signaling pathways in OCM1 cells. OCM1 cells were treated with 0, 0.3, 1, and 10 μM cinobufagin for 24 h. **(A)** The expression of cleaved caspase-3, cleaved caspase-9, cleaved poly(ADP-ribose) polymerase (PARP), and Bcl-2 was detected by Western blot. β-Actin was used as a loading control. The expression of **(B)** cleaved caspase-3, **(C)** cleaved caspase-9, **(D)** cleaved PARP, **(E)** Bcl-2, **(F)** Bcl-xL, **(G)** Bad, and **(H)** Bax was quantified using ImageJ software. The results are shown as the mean ± SEM of three independent experiments [*P < 0.05, **P < 0.01, and ***P < 0.001 compared with the DMSO control].

Next, to determine whether cinobufagin influences the apoptosis of OCM1 cells, we utilized the Annexin V-FITC/PI staining method according to a previous study (16, 17). Our results showed that cinobufagin could induce OCM1 cell apoptosis with apoptosis rates of approximately 12.6, 56.4, and 63.6% after treatment with different concentrations of cinobufagin (0.3, 1, and 3 μM), while the percentage of apoptotic cells showed no statistically significant difference at a concentration of 0.3 μM compared with that in the control group (Figures 3A,B). Furthermore, Western blot analysis showed that cinobufagin significantly upregulated the expression of cleaved caspase-3, cleaved caspase-9, and cleaved PARP in a dose-dependent response (Figures 4A,B,D,E). The mitochondrion-dependent apoptosis pathway is controlled by B-cell lymphoma 2 (Bcl-2) family proteins (18). Antiapoptotic Bcl-2 and Bcl-xl are important in determining cell death. The low levels of Bcl-2 and Bcl-xl could attenuate the inhibitory effect on apoptosis with subsequent induction of the caspase cascade. Here, we observed decreased expression levels of Bcl-2 and Bcl-xl and increased expression levels of proapoptotic proteins Bad and Bax in cinobufagin-treated OCM1 cells (Figures 4A,C,F–H). These data demonstrated that cinobufagin induced cell apoptosis in OCM1 cells via a mitochondrion-dependent pathway.

### Cinobufagin Induced Mitochondrial Membrane Potential Depolarization in OCM1 Cells

**Figure 5:**
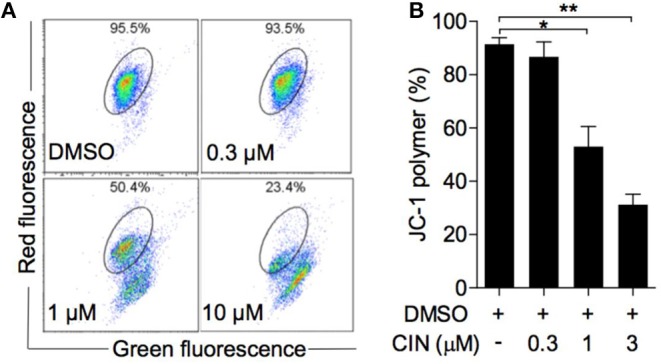
Cinobufagin reduced the mitochondrial membrane potential (MMP) of OCM1 cells. **(A)** OCM1 cells were treated with cinobufagin at 0, 0.3, 1, and 10 μM for 24 h. Then, OCM1 cells were stained with JC-1, and the change in MMP was measured by flow cytometry. **(B)** The percentage of aggregated JC-1 was quantified using GraphPad. The results are shown as the mean ± SEM of three independent experiments [*P < 0.05 and **P < 0.01 compared with the DMSO group].

The alteration of mitochondrial membrane potential (MMP) is one of the intracellular events that occurs in apoptosis (19). JC-1 is an ideal fluorescent probe for detecting MMP. As shown in Figures 5A,B, cinobufagin could decrease the proportion of polymer using the JC-1 indicator. Statistical analysis showed that this effect was dose dependent, with JC-1 aggregates significantly decreasing to 50.4 and 23.4% in OCM1 cells treated with 1 and 3 μM cinobufagin, respectively. The results above demonstrated mitochondrion-mediated cell apoptosis in cinobufagin-treated OCM1 cells.

### Cinobufagin Inhibited Xenograft Growth by Inducing Cell Proliferation

**Figure 6:**
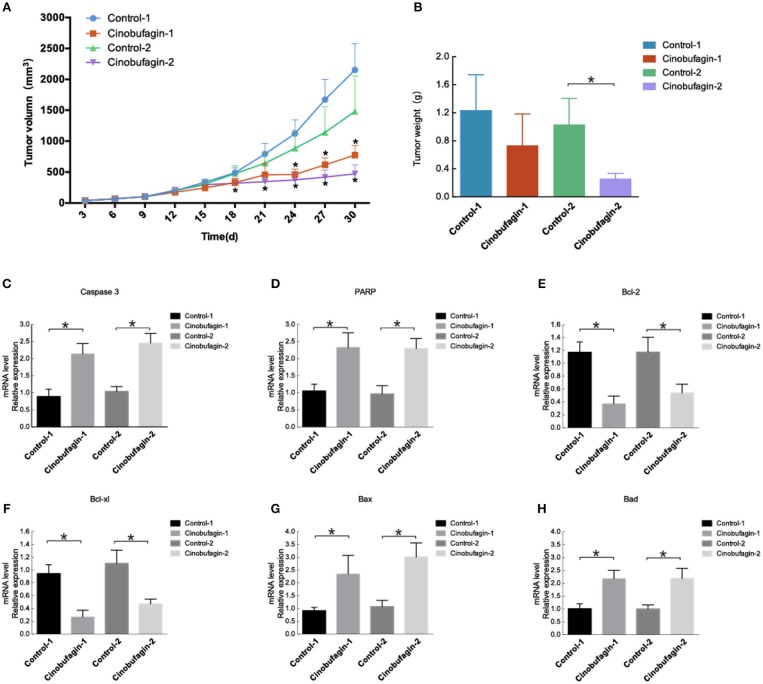
Cinobufagin modulates tumor growth and apoptosis. **(A)** Nu/Nu nude mice were injected with OCM1 cells. After the tumor size reached 100 mm^3^, the experimental group was intraperitoneally injected with cinobufagin at 5 mg/kg once a day for 10 days (cinobufagin 1). The control group (control 1) was intraperitoneally injected with 0.5 mg/kg saline for 10 days. After the remaining 10 mice had tumors up to 300 mm^3^, the tumors were injected with cinobufagin at 5 mg/kg once a day for 7 days (cinobufagin 2). The control 2 group was untreated (control 2). Tumor volume was measured every 3 days. **(B)** After 30 days, the mice were killed, and the tumors were removed and analyzed. The tumors treated with cinobufagin weighed less. The expression of **(C)** caspase-3, **(D)** PARP, **(E)** Bcl-2, **(F)** Bcl-xL, **(G)** Bad, and **(H)** Bax was quantified by RT-qPCR in the tumors of the mice. The results are shown as the mean ± SEM of five independent experiments (*P < 0.05).

Untreated OCM1 cells were injected into nude mice. On the ninth day, the tumors in groups treated with intraperitoneal injection of cinobufagin and direct injection with cinobufagin grew more slowly than those treated with intraperitoneal injection of saline or untreated (Figure 6A). After intraperitoneal injection of cinobufagin in nude mice for 10 days, tumors in nude mice grew more slowly than those treated with intraperitoneal injection of saline at 24 days. Tumors treated with direct injection for 7 days grew more slowly than those in the control group at 18 days. After 30 days, the mice were executed, and the tumors were removed. The tumors from groups directly injected weighed less than those in the control group (control 2), while differences in the groups treated with intraperitoneal injection of cinobufagin were not statistically significant (Figure 6B). RT-qPCR experiments were conducted on the removed tumors. The expression of caspase-3 and PARP was increased in tumor tissues (Figures 6C,D). There was decreased Bcl-2 and Bcl-xl expression in mouse tumor tissues and increased expression of Bad and Bax (Figures 6E–H). These data indicate that proliferation is impaired after cinobufagin treatment *in vivo*.

## Discussion

At present, neoadjuvant chemotherapy for advanced UM have been adopted largely. However, side effects of chemotherapy and drug resistance cannot be avoided (11). Therefore, it is crucial to identify new chemicals targeting malignant cells. Recently, various novel drugs have been detected. Cinobufagin, secreted by the Asiatic toad *B. gargarizans*, is a traditional Chinese medicine that has received increasing attention due to its various biological functionalities (20). Whatever, the role of cinobufagin in UM has not been investigated. Therefore, we performed research on whether cinobufagin could inhibit the proliferation and detected the underlying biochemical mechanism in uveal melanoma cells.

Previous researches have demonstrated that cinobufagin exerts antitumor activities by disrupting the cell cycle progression, inducing cell apoptosis, inhibiting angiogenesis, and reversing multidrug resistance (20, 21). However, to date, the role of cinobufagin in OCM1 is not well-understood. To determine the functions of cinobufagin on OCM1 cells, we measured the antitumor effect of cinobufagin via a colony formation assay. The results demonstrated that cinobufagin inhibit cell proliferation in a dose-dependent manner in OCM1 cells. In addition, G2/M phrase was arrested, and cell apoptosis was enhanced with higher expression of mitochondrion-related apoptotic proteins after cinobufagin treatment in OCM1 cells. Furthermore, loss of MMP was observed to verify mitochondrion-based cell apoptosis.

Cell apoptosis, also known as programmed cell death, initiates by certain internal or external signals. Apoptosis plays crucial roles in aging, neoplasm, and neurological disorders, such as amyotrophic lateral sclerosis and Parkinson's disease (22), and is characterized by chromatin condensation, subsequent DNA cleavage, and shedding of small fragments from cells (23). Two different pathways are involved in the induction of apoptosis, which occur via the mitochondria (the intrinsic pathway) and/or the activation of death receptors (the extrinsic pathway). Both pathways converge to activate caspases as final executioners of cell death (23). The initialization of apoptosis is a powerful therapeutic strategy in the treatment of various cancers (24). Here, flow cytometry analysis of Annexin V-FITC/PI double-labeled cells revealed that apoptotic rates increased in a concentration-dependent manner after cinobufagin treatment in OCM1 cells. Consistent with our findings, cinobufagin induces apoptosis in a wide range of human tumor cells. Combined, these results confirmed that cinobufagin could induce apoptosis in OCM1 cells.

The mitochondrial apoptotic pathway encompasses the production of ROS, loss of ΔΨm, then triggers the release of cytochrome c from mitochondria into cytoplasm. The upregulated expression of ROS damages mitochondrial DNA (mtDNA), followed by transcriptional impairment of mtRNAs relevant to the respiratory chain, further enhancing ROS generation (25). Excessive ROS production and oxidative damage to mitochondrial membrane trigger apoptosis and other a series of mitochondrion-associated biological events (26, 27). The Bcl-2 family proteins governs the intrinsic mitochondrial apoptotic pathway (18). Antiapoptotic Bcl-2 and Bcl-xl are important in determining cell fate, and downregulated expression of Bcl-2 causes the loss of ΔΨm and the subsequent release of cytochrome c from the mitochondrion into the cytoplasm (28). The proapoptotic proteins Bad and Bax can reside in the cytoplasm, translocate to the mitochondria after receiving death signals, and promote the release of cytochrome c in mitochondria. Cytochrome c then binds to Apaf-1, which activates caspase-9 to start the caspase cascade where caspase-3 is cleaved, which triggers the cleavage of numerous key cellular substrates, such as PARP, leading to the fragmentation of DNA and thus inducing cell apoptosis (23). Our results showed the decreased antiapoptotic protein Bcl-2 and Bcl-xl and increased proapoptotic proteins Bad and Bax after treated with cinobufagin in a dose-dependent manner, accompanied with gradual expression of cleaved caspase-9, PARP, and caspase-3 in OCM1 cells.

Animal experiments further demonstrated that cinobufagin could significantly inhibit tumor growth. After 24 days, tumors in nude mice grew more slowly after 10 days of treatment with intraperitoneal injection of cinobufagin than after treatment with intraperitoneal injection of saline. However, compared with control groups, tumors with direct injection for 7 days grew more slowly on the 18th day. Direct injection of cinobufagin into tumors in nude mice was more effective than intraperitoneal injection of cinobufagin when the cinobufagin was at the same concentration. The mice were killed after a treatment duration of 30 days, and the tumors were excised. The directly injected tumors weighed less than the tumors in the control group (control 2), while the tumors treated with intraperitoneal injection of cinobufagin were not significantly different by weight; the concentration of cinobufagin may need to be increased, or there may not have been enough time for cinobufagin to function after intraperitoneal injection. RT-PCR experiments were conducted on the removed tumors. Increased expression of caspase-3, PARP, Bad, and Bax was detected in the tumor tissues. However, compared to control tissues, the expression of Bcl-2 and Bcl-xl were decreased in mouse tumor tissues. These data indicate that proliferation is impaired after treated with cinobufagin *in vivo*.

Furthermore, we assessed the decreased ΔΨm after cinobufagin treatment. It was supported by the fact that JC-1 aggregate fluorescence waned in cinobufagin-treated OCM1 cells in a dose-dependent manner. However, there were no significant changes in cell apoptosis when treated with 0.3 μM cinobufagin. Bcl-2 and cleaved PARP expression were not significantly changed at 0.3 μM. This means that the mitochondrial/intrinsic pathway was not activated after treatment with 0.3 μM cinobufagin. As the caspases were activated at 0.3 μM, the extrinsic pathway was activated at lower concentrations, which needs further exploration. In general, our data demonstrated that cinobufagin first activates the intrinsic apoptosis pathway to induce OCM1 cell apoptosis.

## Conclusion

In conclusion, this study revealed the potential antitumor role of cinobufagin in treating UM and encourages further anticancer research on cinobufagin, which could lead to further studies of cinobufagin for clinical usage in UM.

## Data Availability Statement

All datasets generated for this study are included in the article/Supplementary Material.

## Author Contributions

LF and LZ contributed conception, design of the study, and wrote sections of the manuscript. LF, TG, HW, and LZ organized the database. LF, TG, HF, and LZ performed the statistical analysis. LZ wrote the first draft of the manuscript. All authors contributed to manuscript revision, read, and approved the submitted version.

### Conflict of Interest

The authors declare that the research was conducted in the absence of any commercial or financial relationships that could be construed as a potential conflict of interest. The handling Editor declared a shared affiliation, though no other collaboration, with all the authors.
