# Supplementary material for: Study of Cinobufagin as a Promising Anticancer Agent in Uveal Melanoma Through Intrinsic Apoptosis Pathway
Source: Front Oncol. 2020 Apr 2;10:325. doi: 10.3389/fonc.2020.00325 (PMC7142239; doi:10.3389/fonc.2020.00325)
Supplement: Supplementary file 1 [file Table_1.docx]

| Supplemental Table 1. Primers used in this study | |
| --- | --- |
| Primer name | Sequence (5’- 3’) |
| *caspase-3-*F | GGACTGTGGCATTGAGACAG |
| *caspase-3*-R | CGACCCGTCCTTTGAATTTC |
| *PARP*-F | TGGAAAAGTCCCACACTGGTA |
| *PARP*-R | AAGCTCAGAGAACCCATCCAC |
| *Bcl-2*-F | ATGTGTGTGGAGAGCGTCAA |
| *Bcl-2*-R | CAGGAGAAATCAAACAGAGGC |
| *Bcl-xl*-F | GAATCTTATCTTGGCTTTGGA |
| *Bcl-xl*-R | GTAGAGTGGATGGTCAGTGT |
| *Bax*-F | GGTTTCATCCAGGATCGAGACGG |
| *Bax*-R | ACAAAGATGGTCACGGTCTGCC |
| *Bad*-F | TCCCAGAGTTTGAGCCGAGT |
| *Bad*-R | ATGTGGAGCGAAGGTCACTG |
| *GAPDH*-F | AGGTCGGAGTCAACGGATTTG |
| *GAPDH*-R | TGTAAACCATGTAGTTGAGGTCA |
